# Rectal Leiomyosarcoma Treated With Surgery and Adjuvant Radiotherapy: A Case Report With a Brief Review of Radiotherapy Techniques From a Radiation Oncologist’s Perspective

**DOI:** 10.7759/cureus.89843

**Published:** 2025-08-11

**Authors:** Seok Ho Lee, Seung Yeon Ha, Jeong-Heum Baek

**Affiliations:** 1 Radiation Oncology, Gil Medical Center, Gachon University College of Medicine, Incheon, KOR; 2 Pathology, Gil Medical Center, Gachon University College of Medicine, Incheon, KOR; 3 Surgery, Gil Medical Center, Gachon University College of Medicine, Incheon, KOR

**Keywords:** 3d conformal radiotherapy, gastrointestinal stromal tumor, radiotherapy, rectal leiomyosarcoma, ultralow anterior resection

## Abstract

We report the case of a 58-year-old male who presented with diarrhea, fever, and weight loss, and was ultimately diagnosed with rectal leiomyosarcoma (LMS). During colonoscopy, a 4 cm polypoid mass was observed 6 cm above the anal verge, with characteristics suggestive of a gastrointestinal stromal tumor (GIST). Colorectal magnetic resonance imaging showed about a 4.1 x 3.3 x 3.3 cm-sized pedunculated mass in the mid-rectum with two possible pathologic lymph nodes. A pathological review of colonoscopic biopsy revealed atypical spindle cell proliferation, suggestive of GIST. Based on this, the initial impression of the patient was a GIST, and the patient underwent ultralow anterior resection. Microscopically, there was a proliferation of spindle cells. Immunohistochemical stain showed that tumor cells co-expressed actin, desmin, and caldesmon, whereas S100 protein, CD34, and DOG-1 were negative. Based on these findings, the final pathological diagnosis was LMS. The patient received postoperative adjuvant radiotherapy (RT) at a dose of 54 Gy. To date, the exact role, specific indications, and techniques of RT, including the RT field and dose/fractionation for rectal LMS, have not yet been clearly defined. Herein, we present a case of rectal LMS treated with adjuvant RT, accompanied by a brief review of RT techniques reported to date for this rare entity.

## Introduction

Leiomyosarcoma (LMS) is a rare, aggressive tumor originating from smooth muscle cells. It represents less than 0.1% of all colorectal malignancies [1,2]. LMS can be misdiagnosed as a gastrointestinal (GI) stromal tumor (GIST), the most common type of mesenchymal neoplasm of the GI tract [3]. Although the prognostic indicators are different between these two types, the clinical courses and prognoses are quite similar, and complete resection is the principal treatment for primary GIST and LMS [4]. LMS has a high prevalence of recurrence and aggressiveness, which results in poor prognosis depending on the tumor site [2]. In rectal LMS, the optimal additional treatment approach is yet to be determined [5], although the principal treatment is surgery, such as in primary rectal GIST. In this study, we analyzed the pathological findings, including immunostaining and imaging findings, specifically magnetic resonance imaging (MRI) and positron emission tomography (PET). Based on these findings, we present the case of a 58-year-old man diagnosed with rectal LMS who was treated with surgery followed by adjuvant radiotherapy (RT). We also briefly review the major issues related to rectal LMS reported worldwide, focusing on insights from the perspective of a radiation oncologist. 

## Case presentation

**Figure 1:**
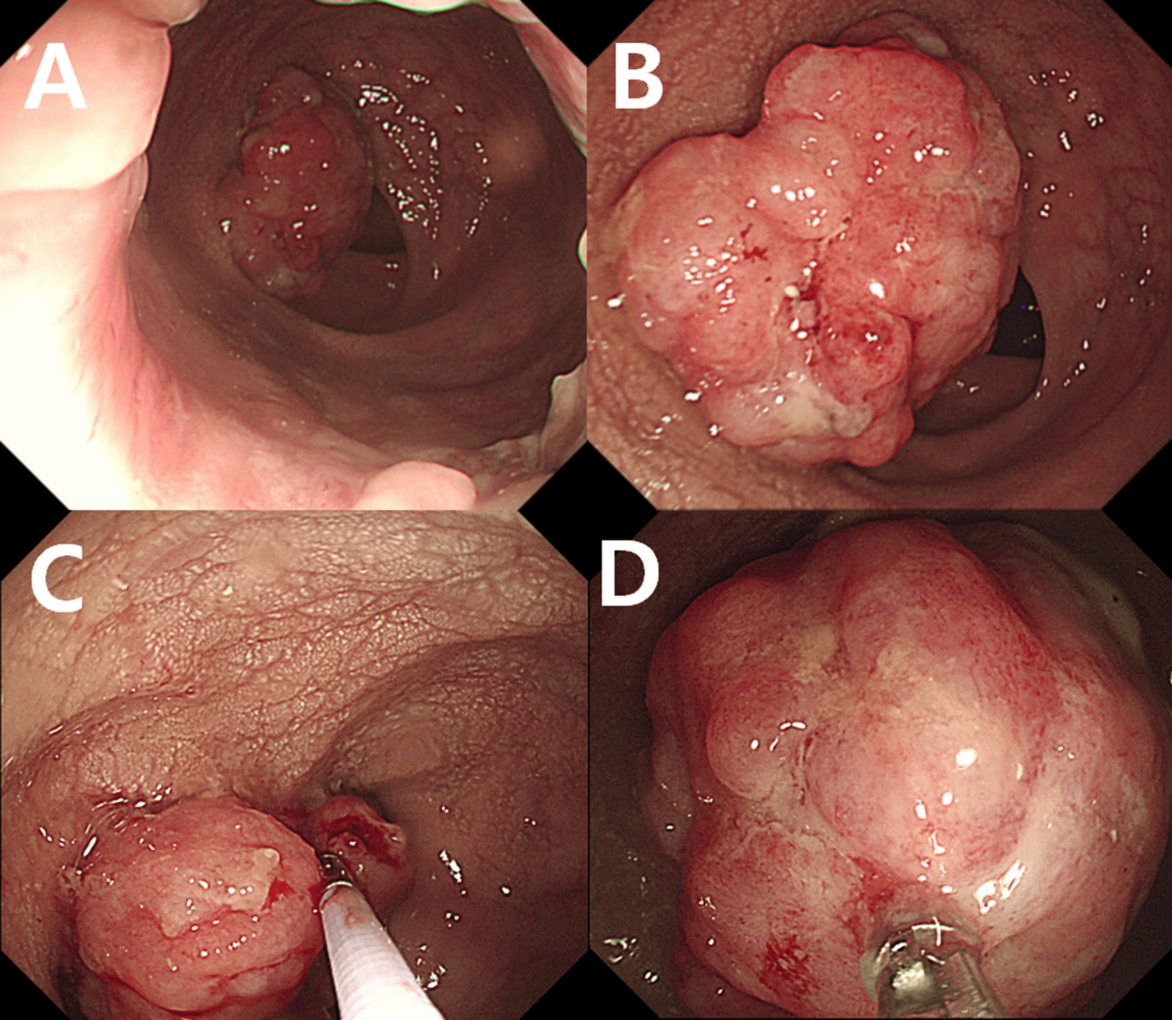
Colonoscopic findings. (A and B) Colonoscopic examination revealed a polypoid mass located approximately 6 cm proximal to the anal verge, measuring approximately 4 cm in maximal diameter. (C and D) The lesion’s gross features were suggestive of a gastrointestinal stromal tumor, and a pathological biopsy was subsequently performed to obtain histopathological confirmation.

A 58-year-old man presented with diarrhea, fever, and weight loss over the previous two months. The patient was referred to our clinic because these symptoms had progressed even though he had been taking medication prescribed by the local clinic. Social history revealed a 10-pack-year history of smoking. Family history was significant for a paternal uncle with gastric cancer. Occupational history included 15 years of employment as a taxi driver. This patient had no known medical history; however, 20 years ago, he underwent a gastrectomy for gastric lymphoma, followed by adjuvant RT. Following the evaluation, the patient was scheduled to undergo a colonoscopy. An approximately 4-cm-sized polypoid mass, located approximately 6 cm proximal to the anal verge (AV), was identified via colonoscopy. The lesion’s gross features suggested a GIST, and a pathological biopsy was subsequently performed (Figure 1).

**Figure 2:**
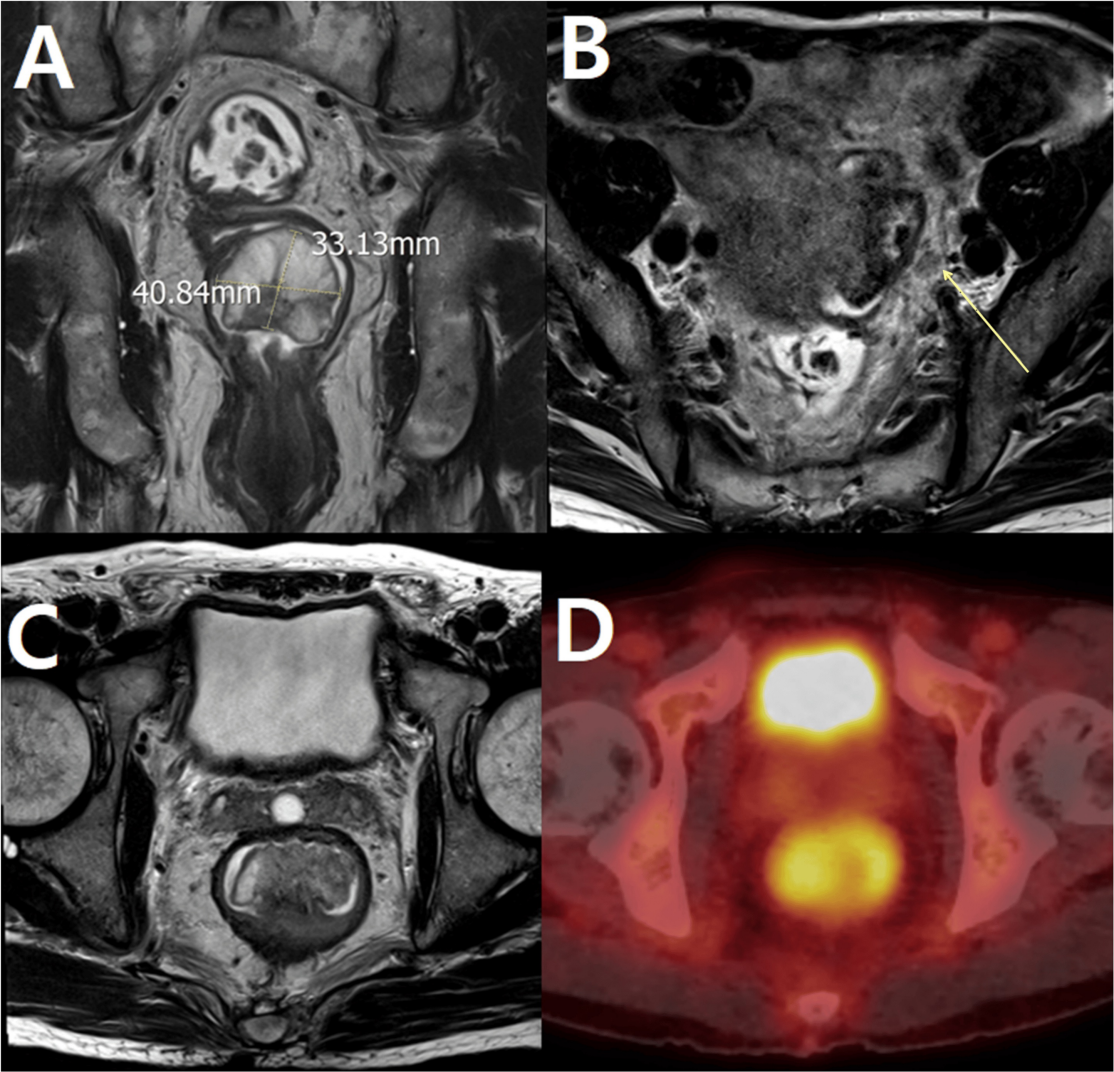
MRI and PET/CT findings. About 4.1 × 3.3 cm pedunculated mass with internal T2 high signal intensity (SI) in the posterior wall of the mid-rectum (4-8 hour direction) (A) with a possible pathological lymph node (arrowed) around the peri-rectum (B). Hypermetabolism is observed on PET imaging at the location of the enhancing mass seen on MRI (C and D). MRI, magnetic resonance imaging; PET, positron emission tomography PET, positron emission tomography

The biopsy results revealed atypical spindle cell proliferation, and the overall findings, including immunohistochemical analysis, suggested the possibility of either a GIST. Follow-up MRI showed a pedunculated mass measuring approximately 4.1 × 3.3 cm with internal T2 high signal intensity in the posterior wall of the mid-rectum (corresponding to the 4-8 o'clock position), along with a possibly pathological lymph node around the perirectal area (Figure 2). The outer muscularis propria of the rectum was preserved without evidence of disruption. Following PET imaging, hypermetabolic activity suggestive of a pathologic lesion was observed in the rectal lesions identified on MRI (Figure 2).

**Figure 3:**
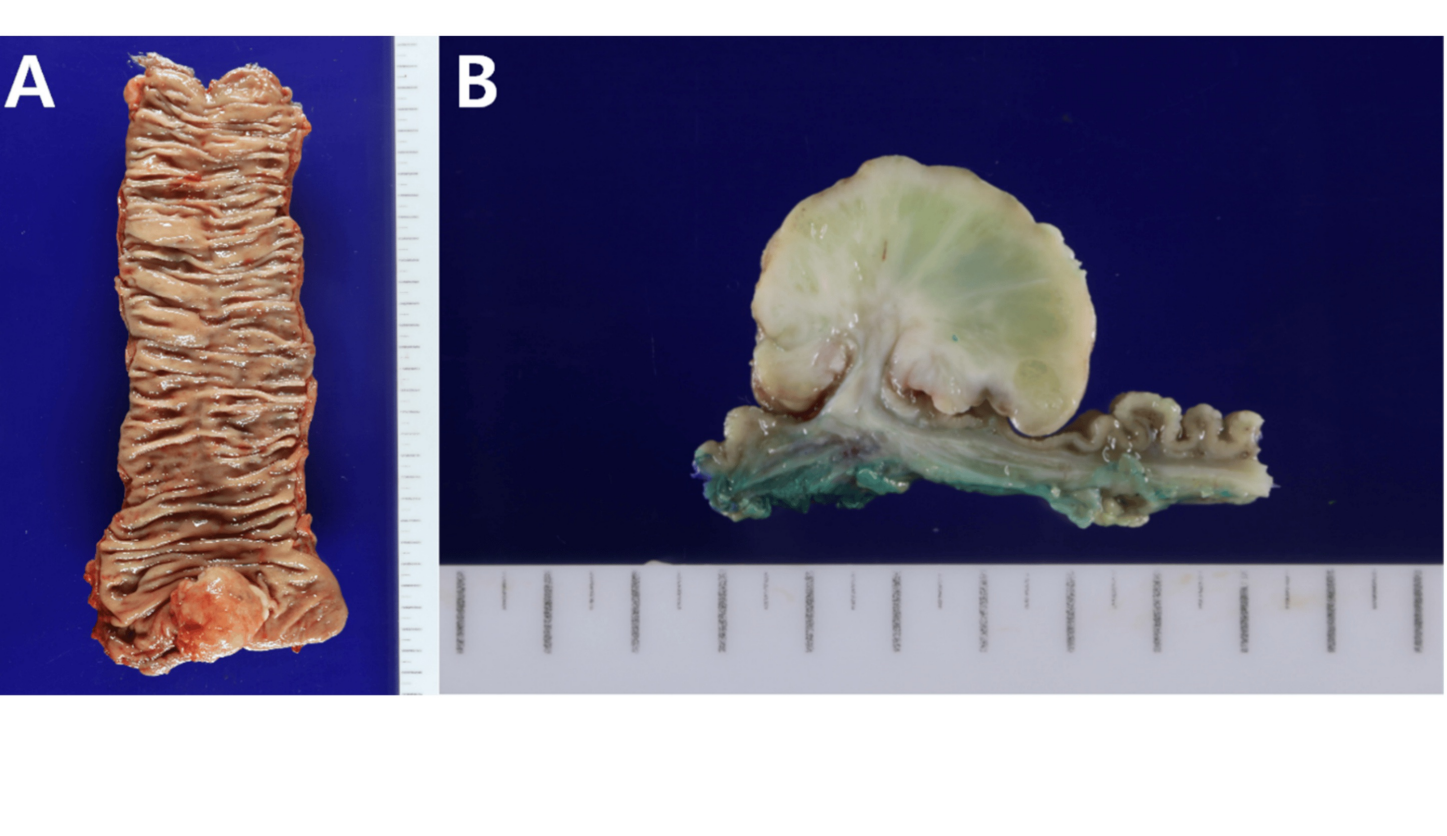
Gross findings of the tumor. (A) Low anterior resection of the rectum shows a polypoid mass. The tumor measured 4.5 × 4.5 cm. (B) The cut surface of the tumor reveals a whitish, fish-flesh appearance, with no evidence of hemorrhage or necrosis.

However, the lymph node lesions that were suspicious for metastasis on MRI did not show increased uptake on PET. An ultralow anterior resection (uLAR) with regional lymph node dissection was performed, and a protective loop ileostomy was constructed to divert fecal flow and protect the anastomosis. During surgery, the mass was located 6 cm from the AV and measured approximately 4 cm in size with a stalk-like appearance. On visual inspection, it appeared to be a malignant GIST. After uLAR, the gross findings showed a polypoid mass measuring about 4.5 cm x 4.5 cm, with a cut surface revealing a whitish fish fresh appearance that was neither hemorrhage nor necrosis (Figure 3).

**Figure 4:**
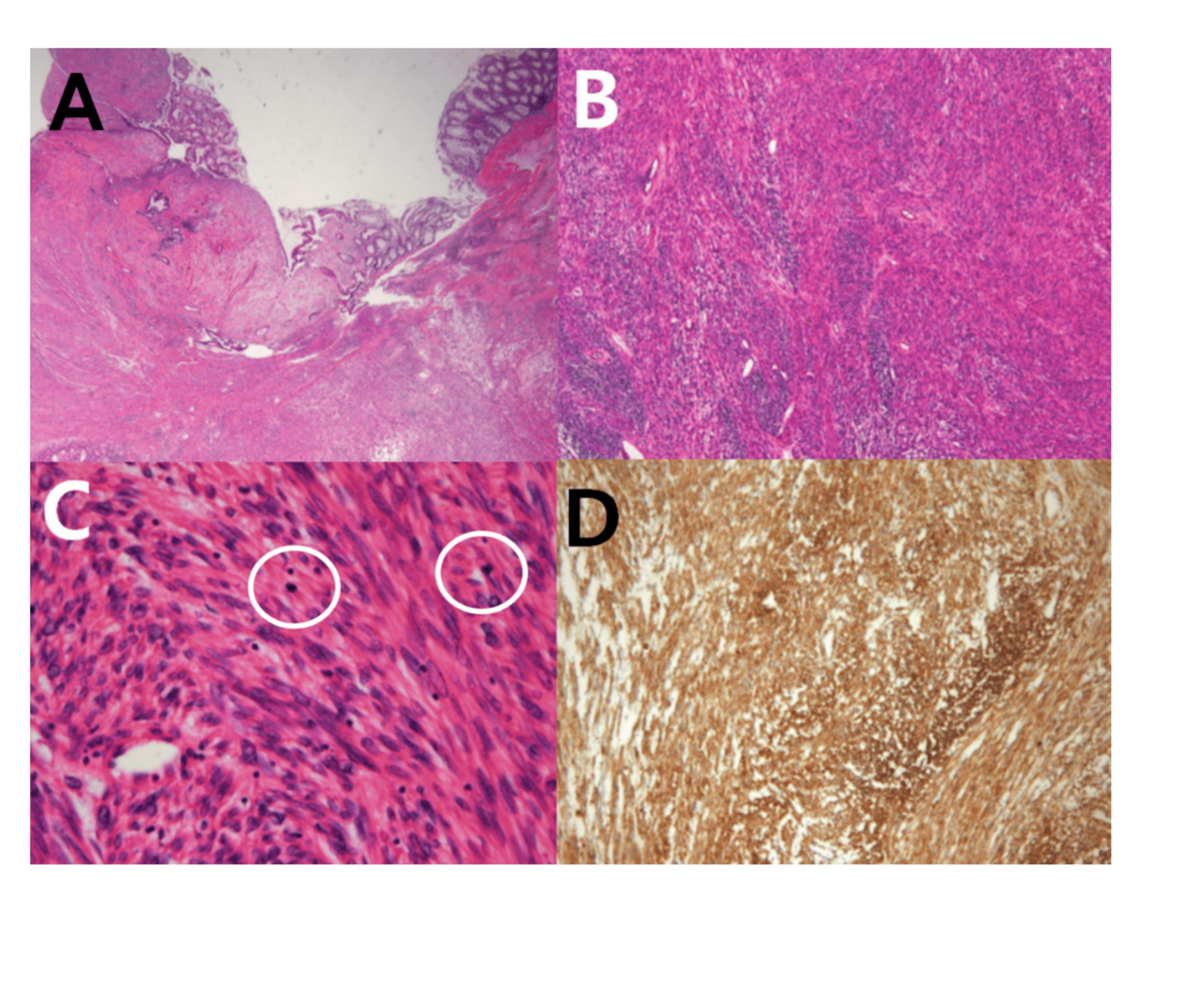
Microscopic findings. (A) The tumor is located in the submucosa and muscularis propria, with overlying mucosal ulceration. (B) Hematoxylin and eosin (H&E) staining shows a proliferation of spindle cells with eosinophilic cytoplasm. (C) On high-power view, several mitotic figures are observed (highlighted with white circles). (D) The tumor cells are positive for α-smooth muscle actin.

Microscopic findings revealed mucosal ulceration and proliferation of atypical spindle cells with eosinophilic cytoplasm. On MRI, two perirectal lymph nodes were suspected of metastasis; however, pathological examination revealed no evidence of metastasis in any of the 57 resected lymph nodes (0/57). There was high mitotic activity (14 mitoses) per 10 high-power field (HPF). Immunohistochemical stains showed that the tumor cells expressed α-smooth muscle actin (Figure 4), desmin, and caldesmon, whereas S100 protein, CD34, and DOG-1 were negative.

**Figure 5:**
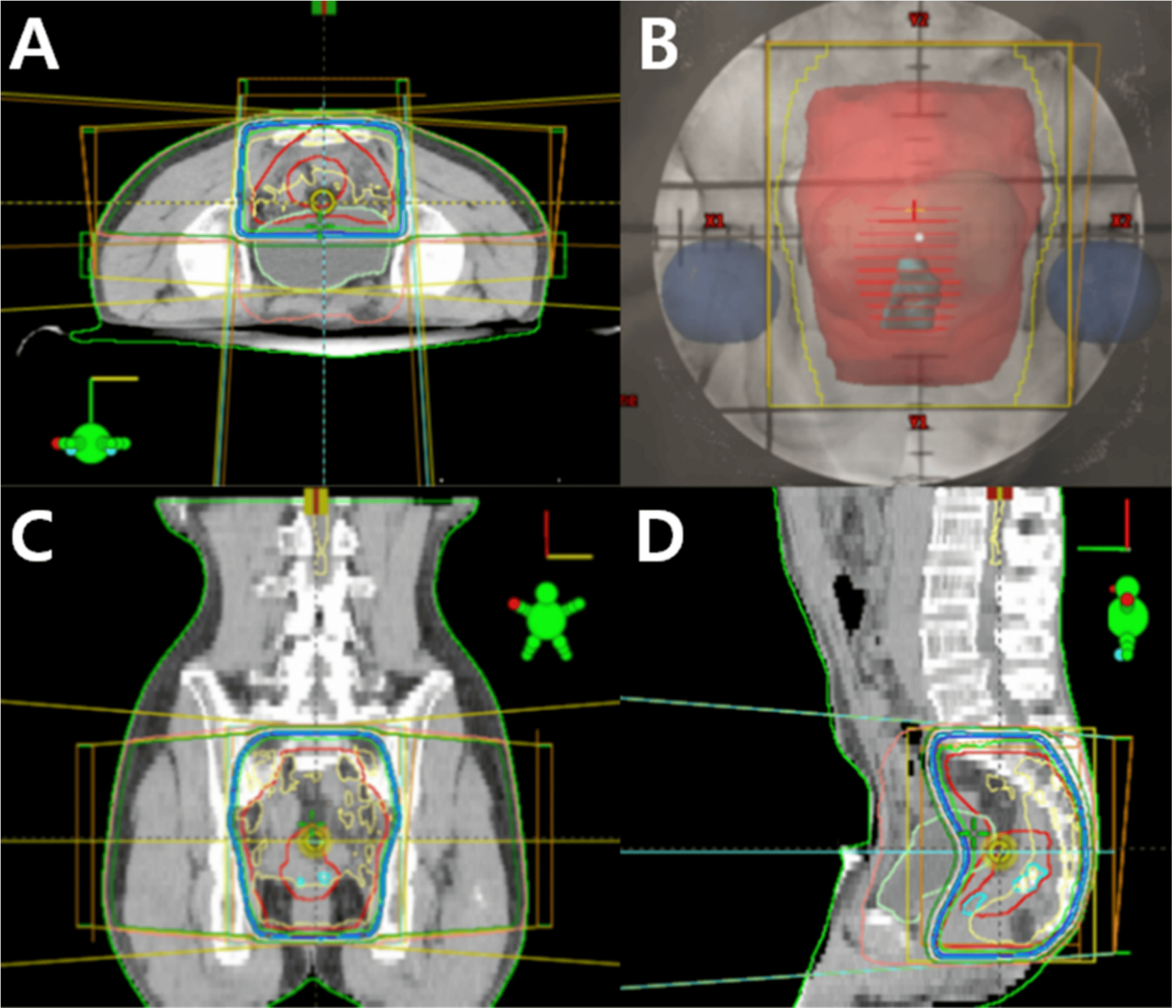
Adjuvant radiotherapy planning. Initially, pelvic radiotherapy was performed using three-dimensional planning with a three-field technique (posterior, left lateral, and right lateral). (A) Axial view, (B) posterior beam’s-eye view, (C) coronal view, (D) sagittal view. The total prescribed dose was 54 Gy, delivered in 1.8 Gy per fraction. A radiotherapy boost to the tumor bed was planned at 45 Gy.

Based on these findings, the final pathological diagnosis was LMS, with a histological grade of 2. On pathological examination, the safe distal margin was 0.8 cm. There was no involvement of the proximal, distal, or circumferential margins. Based on these findings, the final pathologic diagnosis was LMS with histological grade 2. According to the AJCC 8th edition staging system for intra-abdominal or retroperitoneal sarcomas, the pathologic stage of this patient is T1N0M0 with a histologic grade of 2, corresponding to AJCC stage IIA. Based on the FNCLCC grading system [6], the tumor received 2 points for differentiation, 2 points for mitotic count, and 0 points for necrosis, resulting in a total score of 4 points, corresponding to FNCLCC grade 2 (intermediate grade). The postoperative treatment plan was determined by a multidisciplinary team, and adjuvant RT was selected by treatment guidelines for abdominal/retroperitoneal sarcomas. This decision was based on the surgical findings at the time of the operation and postoperative pathological features, including a short resection margin, tumor differentiation of grade 2, and mitotic activity. However, chemotherapy was not administered as it was deemed not to affect postoperative histologic grade 2. The patient received postoperative adjuvant RT at a dose of 54 Gy (Figure 5).

In this case, three-dimensional conformal radiotherapy (3D-CRT) was applied as postoperative radiation therapy. CT simulation for RT planning was performed with the patient in the prone position. Imaging was acquired under a bladder filling protocol, which involved either refraining from voiding for at least two to three hours before the scan or, if voiding had occurred, drinking sufficient water before the CT to ensure adequate bladder distention. The RT field and dose were determined by the adjuvant RT for rectal cancer. The clinical target volume (CTV) encompassed the postoperative tumor bed (resection cavity and surgical clips) with a generous safety margin (approximately 3-4 cm margin in the longitudinal (cranio-caudal) direction and about 1.5 cm radially (axially) around the preoperative gross tumor volume or tumor bed) to account for microscopic disease spread according to the Radiation Therapy Oncology Group (RTOG) guideline [7]. Any MRI-visible peritumoral edema or signal change on T2-weighted images is included in the CTV. The pre-sacral space at the level of the tumor bed and any remaining mesorectal tissues should be encompassed in the CTV. A uniform margin (0.5~1cm) is added to the CTV to form the planning target volume (PTV), accounting for setup error and internal motion. Elective lymph node regions (e.g., internal iliac, presacral nodes) are not included in the CTV but in the PTV.

In particular, the pelvic region was determined based on pathological and PET findings. The specific radiation dose was 1.8 Gy per fraction, with a total of 45 Gy delivered to the PTV. An additional 9 Gy boost was administered to the CTV, resulting in a total radiation dose of 54 Gy. No special symptoms or signs were observed during RT. During RT, the patient did not report any acute adverse toxicities such as diarrhea, abdominal discomfort, nausea, dysuria, urinary frequency, or radiation dermatitis of RTOG/EORTC (European Organization for Research and Treatment of Cancer) grade 1 or higher [8], and CT scans at six-month intervals during the 15-month follow-up period were unremarkable. During the follow-up period, there was no evidence of recurrence or metastasis, and no chronic toxicities related to RT were observed.

## Discussion

Here, we report a case of rectal LMS treated with surgery followed by adjuvant RT. In the initial diagnostic approaches for our case, the diagnosis was suspected to be GIST based on colonoscopic findings. The differentiation of LMS from benign leiomyomas is possible based on cellular atypia and mitotic activity. Cellular atypia and high mitotic activity (>10 per 10 HPF) strongly support LMS [5]. In our case, all immunohistochemical markers for the confirmation of LMS were positive and met the above criteria. This result emphasizes the importance of immunohistochemistry in achieving an exact diagnosis of LMS and that good therapeutic outcomes are crucial.

Regarding his past medical history, the patient underwent a total gastrectomy 27 years ago. At that time, early gastric cancer (type IIc) was identified on the posterior wall of the upper body of the stomach during a routine health screening, and a concurrent low-grade B-cell lymphoma (LGBL) was also detected. The surgical resection revealed positive margins, and 5 of the 28 regional lymph nodes were positive for LGBL. Consequently, adjuvant therapy was initiated, consisting of three cycles of CHOP chemotherapy (cyclophosphamide, doxorubicin, vincristine, and prednisone), followed by RT delivering a total of 39.6Gy in 22 fractions of 1.8 Gy each, and then an additional three cycles of CHOP chemotherapy. The RT field was delineated to include the preoperative location of the stomach as well as the abdominal lymph node regions, extending from T11 to L2. In the present case, the tumor measured 4 cm in size and was located 6 cm above the anal verge. Although the patient had a prior history of RT, considering the anatomical location of the previous radiation field, a radiation-induced origin was deemed unlikely.

As mentioned previously, the principal treatment for these cases was complete resection. However, the postoperative treatment approach differed. In the case of primary GIST, the principal treatments are surgery and adjuvant imatinib [9]; whereas the standardized treatment protocols are yet to be determined in cases of rectal LMS [2,9].

As a follow-up treatment after surgery for rectal LMS, adjuvant RT can be proposed by extending the management principles to abdominal/retroperitoneal sarcoma [2,9,10]. Specifically, RT is generally indicated in cases in which the resection margin is positive, with a larger tumor size, and factors that increase the likelihood of local recurrence. A recent meta-analysis showed that adjuvant RT was associated with distinct benefits not only for local control but also for survival compared to surgery alone for retroperitoneal sarcoma [10]. Table 1 compares previously reported cases of rectal leiomyosarcoma treated with adjuvant RT to the present case.

**Table 1:** Comparison of cases receiving adjuvant radiotherapy for rectal leiomyosarcoma. RT, radiotherapy; AV, anal verge; APR, abdominoperineal reseaction; AR, anterior resection; LAR, lower anterior resection; NR, not reported; 3D-CRT, three-dimensional conformal radiotherapy; DoD, died of disease

Authors	Sex/Age	Gross finding	Distance from AV (cm)	size (cm)	Initial therapy	Adjuvant therapy (RT technique)	Reasons for RT	RT dose (Gy/fx)	Recurrence (mo.)	Survival outcome (mo.)
Pavelic et al. [11]	Male/60	Mass	NR	1.8x1.3	APR	RT (NR)	NR	NR (NR)	Local recurrence (1.5)	DoD (7.5)
Sahli et al. [12]	Male/30	Mass	6	8x5x3	AR	RT (3D-CRT)	Grade/Tumor size	50/25 (3D-CRT)	No	Alive (6)
Maruzzo et al. [13]	Female/66	Mass	NR	NR	NR	RT (NR)	Positive Margin	NR (NR)	Local recurrence (120)	DoD (140)
Present study	Male/58	Polyp	6	4.1x3.3	LAR	RT (3D-CRT)	Close Margin/Grade/Mitotic activity	54/28 (3D-CRT)	No	Alive (15)

According to recent systematic reviews, the main roles of RT are to reduce the risk of local recurrence, facilitate R0 resection during surgery, and, in some cases, support sphincter-preserving surgery. When comparing the effects based on treatment timing, neoadjuvant RT significantly reduced local recurrence rates. A meta-analysis focusing on retroperitoneal sarcoma also reported that neoadjuvant RT was more effective than postoperative RT (odds ratio (OR) 0.03, *P* = 0.02). In contrast, adjuvant RT has been used in select cases, such as when surgical margins were positive (R1) or when the tumor size was large, but its efficacy in preventing local recurrence was reported to be lower than that of neoadjuvant RT [5]. However, in the present case, surgery was performed first because the preoperative biopsy suggested the possibility of a GIST.

In our case, the surgical and pathologic findings, including a close surgical margin, tumor grade 2, and a relatively high mitotic index, which were expected to increase the risk of local recurrence, were the reasons for administering adjuvant RT by the reported RT indications [2, 14, 15]. In the present case, the postoperative pathological assessment revealed an 8 mm resection margin, and the decision to administer RT was made during the multidisciplinary team meeting based on literature that broadly defines close margins. In addition, the pathological findings of tumor differentiation of grade 2 or higher and a high mitotic index also served as additional factors supporting the decision to administer adjuvant RT [15]. Among the various adjuvant RT techniques, 3D-CRT was selected in this patient due to a range of factors-such as cost, insurance reimbursement, and effectiveness have also been reported in numerous previous studies [16]. In recent years, for neoadjuvant or adjuvant RT for rectal cancer, the intensity-modulated radiotherapy (IMRT) technique has been used to reduce toxicity and increase the RT dose [17]. Although some reports have compared these two techniques for rectal cancer [17, 18], there are no specific reports on RT techniques for rectal LMS. For representative studies comparing these two techniques, a study reported a lower incidence of acute toxicity in locally advanced rectal cancer treated with IMRT [17]. However, recent studies comparing the efficacy of these two techniques have reported that IMRT is not associated with survival benefits over 3D-CRT for rectal cancer [18]. To decrease the local recurrence, neither the appropriate RT technique nor the exact RT dose has been determined because of the rarity of this disease. In our case, the RT dose for rectal LMS is currently adopted based on cases of rectal cancer. The reported RT dose ranges from 45 to 54 Gy, with a daily fraction of 1.8 to 2 Gy [18]. In the context of RT techniques for rectal LMS, Table 2 provides a comparative summary of 3D-CRT and IMRT across various aspects, supported by relevant references.

**Table 2:** Comparison of 3D-CRT versus IMRT in adjuvant radiotherapy for rectal LMS. LMS, leiomyosarcoma

	3D conformal radiotherapy (3D‑CRT)	Intensity‑modulated radiotherapy (IMRT)
Technique [19]	Uniform-shaped beams to target	Modulated beam intensity to conform closely to the tumor shape
Dose Conformity [20]	Less conformal; more dose to normal tissues	Higher conformity; better organ at risk (OAR) sparing
Toxicity Profile [17]	Higher rates of GI/GU toxicity	Fewer acute/late toxicities
Organ-at-Risk Sparing [21, 22]	Limited sparing of bladder, bowel, and femoral head	Improved sparing (lower V30-V45 to bowel)
Planning Complexity [19]	Simple planning	Requires advanced planning & optimization
Clinical Outcome	Adequate local control with wide margins (clinical studies have not yet been identified)	Better local control in cases with close/positive margins (clinical studies have not yet been identified)
Resource Requirement [16]	Cost-effective, widely available	Needs specialized equipment/expertise

Although it has been reported that the prognosis for rectal LMS patients is poor, and they tend to develop early hematogenous metastases [23], as an adjuvant therapy, chemotherapy has not been considered as a standard treatment approach for retroperitoneal sarcoma [10]. This concept is now also adopted for other cases of soft tissue sarcoma, as in our case.

In our case, during the 15 months of surveillance, two follow-up CT scans were performed at six-month intervals, and the CT findings showed no evidence of recurrence or metastasis. As a result, during the follow-up period after the completion of adjuvant RT, there were no acute or chronic toxicities related to RT, although more follow-up periods are needed to elucidate the clinical outcomes. However, due to the single-case nature, rarity of the disease, and short follow-up period, the role of adjuvant RT in rectal LMS remains unclear. We believe that the way to establish both the role of adjuvant RT and the appropriate RT technique for rectal LMS is to cooperate with several institutions in the near future, based on multi-center collaborative studies.

## Conclusions

To date, clear evidence is lacking regarding the exact role, specific indications, and technical aspects of RT for rectal LMS, including target delineation and dose/fractionation. In this study, we present a case of rectal LMS treated with adjuvant RT, accompanied by a brief review of RT techniques reported to date for this rare entity. A multicenter prospective observational study is warranted to establish a clear and evidence-based treatment strategy.
